# Flexible and cost-effective cryptographic encryption algorithm for securing unencrypted database files at rest and in transit

**DOI:** 10.1016/j.mex.2022.101924

**Published:** 2022-11-11

**Authors:** Vaheedbasha Shaik, Dr. Natarajan K

**Affiliations:** Department of Computer Science & Engineering, School of Engineering & Technology, CHRIST (Deemed to be University), Bangalore, India

**Keywords:** Non-datafiles encryption, Database configuration files encryption, Database internal files encryption, New database encryption method

## Abstract

To prevent unauthorized access to the databases and to ensure that the data of the databases is protected from intruders and insiders, the data is being encrypted at the storage locations. The same goal is achieved with Transparent Data Encryption, a feature that can be found in almost all database products. However, it has been observed that the non-datafiles are being ignored and there is no standard encryption for them like there is for datafiles. Moreover, there was no standard algorithm to encrypt them without relying on third-party tools. Therefore,

• This study provides a robust algorithm to perform the encryption. This presentation also describes the importance of non-datafiles encryption, and how some non-datafiles can pose a threat to data and infrastructure without encryption.
• The practical implementation of the non-data file encryption algorithm shows the authentic results. Further, unlike existing algorithms, the proposed algorithm gives the file owner full control over the encryption logic.
• In the encryption process, two levels of encryption logics are combined with a passcode lock, while the same combination of two levels of reversing encryption and passcode is used in the decryption process to convert encoded data back into text format.

## Graphical Abstract

**Figure:**
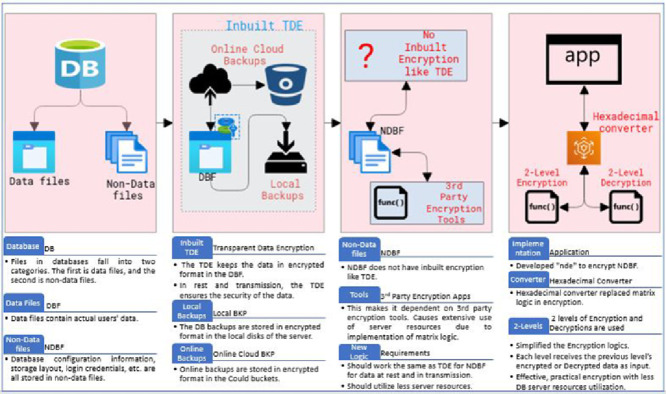

**Specifications table**

**Table:** 

Subject Area:	Computer Science
More specific subject area:	*Encryption of non-database files and configuration files using a simplified algorithm along with practical Implementation.*
Method name:	*2-Levels of Non-Datafiles Encryption (NDE)*
Name and reference of original method:	The original encryption method AES was studied to understand the encryption [30]. The same Hexadecimal coding has been customized with 2 level of encryption process with passcode lock.
Resource availability:	https://github.com/vaheedbashashaik/NDE_Algorithm *The file “nde” is compiled binary executable file. The results can be tested and regenerating using it.*

## Introduction

In recent years, digitalization has accelerated to new level. By using digitalization, the work can be done faster with automation than with a manual work order [1]. Digitalized data will be stored in the data storages according to the storing order, such as relational, graphical, spatial, and multimedia formats in datafiles of databases [2]. The databases always have two types of files, those are datafiles and non-datafiles [3]. The user's data will be stored in the datafiles and the database related data would be stored in the non-datafiles [4,5].

Many concerns over privacy and security are surfaced about user's data storage patterns because a compromised security is always a threat to individual privacy, organizations, and the integrity of the country [6]. In response to these concerns, database software vendors created the transparent data encryption (TDE) technique. TDE is a method that has been adopted by practically all database software vendors, such as Oracle, MySQL, MSSQL, etc. The TDE method not only encrypts data in transit and data in rest but also prevents administrators from viewing it [7,8].

An analysis of TDE and non-TDE databases was conducted in recent studies, which explains the advantages and disadvantages of both methods [7,8]. In the end, TDE is the best choice when it comes to security and privacy. The purpose of this TDE method is to encrypt the datafiles of databases where users' data is to be stored. However, this method isn't designed to encrypt certain non-datafiles in the databases. these non-datafiles will store some very important information too. Oracle databases keep very important information like data file location, names, listener information, and most of the internal database structure in their text parameter files, listener.ora files, and tnsnames.ora files [9]. The ‘my.cnf’ configuration file in MySQL databases contains the details of the database structure and optional parameters such as usernames and passwords [10]. Likewise, the parameter file of MSSQL database stores very crucial information about storages and server details [11]. According to the literature review, there is no encryption method like TDE for non-datafiles. There is a significant research gap in recommending a new encryption method that keeps non-datafiles safe when they are at rest or in transit. In the proposed methodology, a new algorithm has been implemented practically for non-datafiles to be encrypted.

This paper has been presented as an introduction giving an overview of the importance of encryption and a glimpse of a research gap. The next related work section will describe Common files and their categories, as well as the data that would be contained in them. The research methodology explains the proposed algorithm, modules, and pseudo-code that will be implemented. In the results and discussion section, the proof of the proposed algorithm implementation will be shown with actual screenshots from the servers. The future work and conclusion section gives the summarized report about the new scope of the work and complete report of results and the references section provides details with cross-referenced materials.

## The related work

It is common for databases to have two types of files, one of them called datafiles, which are meant to store user data, and the other called non-datafiles [4,5]. The non-datafiles contain information about the databases, including directory structures, locations of datafiles, and their status. All database software vendors have implemented Transparent Data Encryption (TDE) regardless of the data they are going to store [[9], [10], [11]]. Using TDE, all questions and concerns associated with the privacy and security of datafiles will be answered. The process of falsely presenting sensitive data to an unauthorized user is known as data masking. Data masking is one of its features and will prevent even administrators from viewing data. Even from the database command-line interface (CLI) at the command prompt, the select command cannot access the data [12]. In addition to all these features, the TDE can do secure data transfer to other locations, and backups of these databases can be stored on remote devices in encrypted form [13]. Therefore, the TDE method can prevent any third party from analysing the backup files. however, it is not the case with non-datafiles, these files are simply being copied to the backup storages or backup tape drives or cloud buckets in a plain text format. If these backup storage locations are accessible to any unauthorized database administrator or third party, then they can read these files with read access. According to the literature review, there is no proper encryption method that meets the privacy and security standards for non-datafiles. It has been observed that most database binaries like Oracle, MySQL, etc., contain these non-datafiles in a non-encrypted format.

The datafiles of the databases store the data in different formats. Each block of data is the smallest level of granularity that can be stored [14]. A single block will have multiple sections for maintaining both the actual data and the reference to the data. In addition, the internal structure of the block will vary depending on which segment it belongs to [14,15]. The segment is a collection of extents where the layout of storage can be deferred depending on the type of object. In the case of an index segment, the block structure contains information about the physical location of the table row of the respective data segment. If the block is part of the table segment, it contains the actual data as well as metadata about the block and datafiles [14,15]. Even if the data is stored in a different format in a data file, there is a way to view it at the operating system level. The ‘strings’ utility makes it possible to view data even if the file has been formatted differently [16]. Any unauthorized third party or insider who has access to the ‘strings’ utility can view the data from the datafiles in the database [17].

In case of data transfers between two locations, the intruder can hack the connection to gain access to the data. In another case, if the backups are stored on tape backup drives or in any other cloud storage bucket, any user with reading access can view data by using the ‘strings’ utility [17]. Determining the loyalty of authorized staff is usually difficult, sometimes one of the intruders could be authorized personnel. To cope with such scenarios, TDE has been incorporated into every database product. Using the TDE mechanism, encrypted datafiles can display only encrypted data which makes no sense to the reader, usually, there will be a lot of combinations of symbols that are unknown to the reader. The only possible way to read the data file again upon decryption of it with a secured key [17].

Interestingly, this TDE method cannot be used on non-datafiles, although non-datafiles contain a great deal of essential information. To secure the database information completely, non-datafiles must be encrypted as well.

### Perspectives on the non-datafiles

This section discusses the importance of non-datafiles and the justification for encrypting them. To keep their operations running, the databases will maintain some internal files. Maintaining these files is also important to keep the database running smoothly [[18], [19], [20]]. There are some non-datafiles formatted in binary, such as system parameter files; this type of binary structure can provide security up to a remarkable degree. However, there are still many files in text format. Likewise, it is necessary to have an initial configuration file to start up a database. This file is called in different ways in different database software binaries like parameter files or configuration files [[18], [19], [20]].

These configuration files are essential for starting the database in its first phase. Memory structure information, datafiles' locations, and some very important files like control files details are contained in these files that can create an unrecoverable state if they are accidentally deleted [[18], [19], [20]]. Some database binaries contain usernames and passwords in their configuration files, like MySQL. Oracle databases have several very important non-datafiles, apart from the parameter files, such as “listener.ora”, “tnsnames.ora”, and “sqlnet.ora”. When an intruder gets access to the ‘listener.ora’ file, he can find out details about the hostname/IP address, port number, and other accessing points [21]. In the case of Unauthorized Access to ‘tnsnames.ora’, it is possible to view the details of other sources of information and Transparent Network Substrate (TNS) entry details. The sqlnet.ora file can provide information about the type of connections the current database can accept. Therefore, non-datafiles should be encrypted regardless of whether they are in transit or at rest.

### Insights on 3rd party encryption engines

Non-datafiles can be encrypted by using the third-party encryption engines. The non-datafiles can be uploaded to these encryption engines to encrypt them on the source side, and these engines can also be used at the destination to decrypt them. However, some concerns may arise with the use of encryption engines for this purpose.

Every production database must meet the annual compliance requirements. Neglecting compliance audits could also lead to hefty fines from government agencies [22,23]. There are inbuilt features available for data file encryption like TDE which does not require annual compliance [24]. As there is no inbuilt feature for non-data file, all third-party encryption tools must pass compliance tests. Based on the literature review, there are no proper encryption methods for non-datafiles that meet the security and privacy standards [25,26]. Auditing of the databases will be carried out annually to verify their integrity and to identify any vulnerabilities. In the unlikely event, if the third-party encryption software does not comply with the current rule sets of audit operations, then it cannot be continued on the database server and most of the encryption software will not succeed consistently [[27], [28], [29]]. To perform any operations on database servers, the third-party encryption software must comply with the database auditing rules during the audit process evaluation [30]. Moreover, the non-datafiles need to be uploaded into third-party software engines for encrypting and decrypting, where these vendors know the entire logic behind the process. The database companies must rely on third-party encryption software companies; there is no other alternative.

As a solution to eliminating such issues, database software companies developed the TDS method to encrypt datafiles. However, the TDS method does not include the possibility of encrypting non-datafiles. Therefore, a new algorithm is needed for non-datafiles, one that can behave like a TDS and must always satisfy audit and compliance requirements.

## The research methodology

A good defensive configuration for Database servers is always needed to deal with cyber threats. To prevent any type of intruder activity, the database servers have been encrypted in different ways. The TDE technology has been improvised to mask data even from database super users and administrators [24]. The TDE method offers security, privacy, and encryption not only at the database server level but also when storing data remotely. In general, remote storage locations are used for backups in the event of a restore or recovery scenario. The TDE encryption method provides security to these backup files by storing them at remote storage in an encrypted format [24]. Despite this, this TDE encryption method is not applicable to all the files in the databases.

As mentioned earlier, there are two types of files in the databases. The TDE method is intended to encrypt only datafiles, but not any other type of files. In all database products, the non-datafiles stores sensitive information about the database's internal structure. The MySQL database product even has text files that contain information like usernames and passwords. Therefore, there is a need for a new type of authentication-based encryption method for these text files. It should also be able to offer security when the data is in the rest state in remote backup locations.

### The architecture of non-datafile encryption algorithm

It is a results-driven process within the architecture. Results from the previous component will be passed on to the next component to derive the required results. It will be carried out until the end has been reached, either in terms of encryption or decryption, as intended in the operation. It is not necessary for the end users to be the same person in order to encrypt or decrypt the non-datafiles. The proposed architecture focuses on flexibility, as there are no constraints or boundaries that restrain the execution of the code. Any operating system can run the proposed code, regardless of the platform in Unix flavours. Physical server access will be restricted in the cloud environments since these servers are configured from shared resources. However, the specific logical units and authorized components of the configured servers cannot be restricted. Therefore, the logic of encryption methodology can be executed by the reader and can be edited in his or her personal environment to regenerate the results.

To build a Data Center (DC), an enormous amount of investment is required. Serves can be placed in such secure environments by paying rent or lease agreement expenditures. Hosting services in DC environments offers a variety of access options. DC environments offer greater configuration flexibility than cloud environments. Unlike cloud vendors, DC vendors have no restrictions on owned infrastructure. There will be no variations in the results with the same server configuration environment when executing and configuring the proposed architecture in cloud or DC environment. Even though the code is in bash shell scripting, the executable file must be compiled on different endian operating systems. The same executables shouldn't be used on big endian operating systems like Solaris if they are created according to the proposed methodology on small endian Linux platforms. It is possible to create executable files from the proposed logic upon compilation on any platform using a bash shell.

**Fig. 1:**
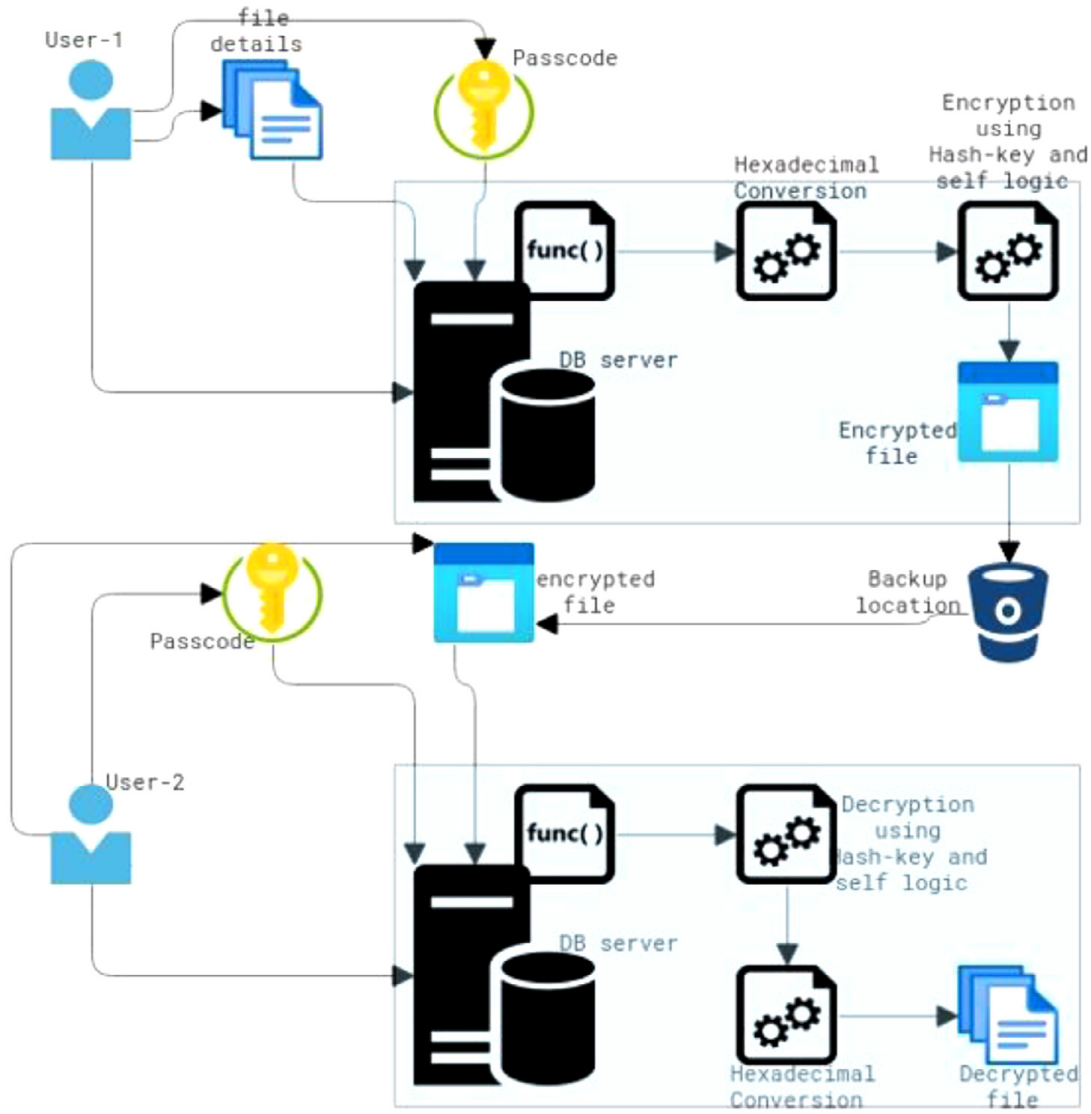
Non-datafile encryption (NDE) algorithm.

In Fig. 1, the components of the non-data file encryption architecture are described. The functionalities of encryption components have been driven by User-1 in Fig. 1. An encryption operation would be carried out in a database server to encrypt non-datafiles by the proposed logic. The logic transforms the non-datafile text into the hexadecimal code and then encrypts it several times with a hash key. The encrypted file will then be moved to remote backup locations, such as tape drives, storage buckets, and disk *drives.*

The functionalities of decryption components have been driven by user 2 in the Fig. 1. According to the proposed logic, a decryption operation would be carried out in the database server to decrypt non-datafiles. After completing a couple of decryption operations, the logic converts the non-datafile's hexadecimal code into text format. In the final step, the encrypted file will be converted into a human-readable text format.

In both the logic of encryption and descriptions, the passcode works as a combination lock for the text. If the user provides the wrong password, the decryption process will not show the actual test rather it shows the unknown symbols to the insider or intruder. Thus, the passcode provides the combination locking and unlocking mechanism to the non-datafiles.

### The design and process flow of non-datafile encryption algorithm

**Fig. 2:**
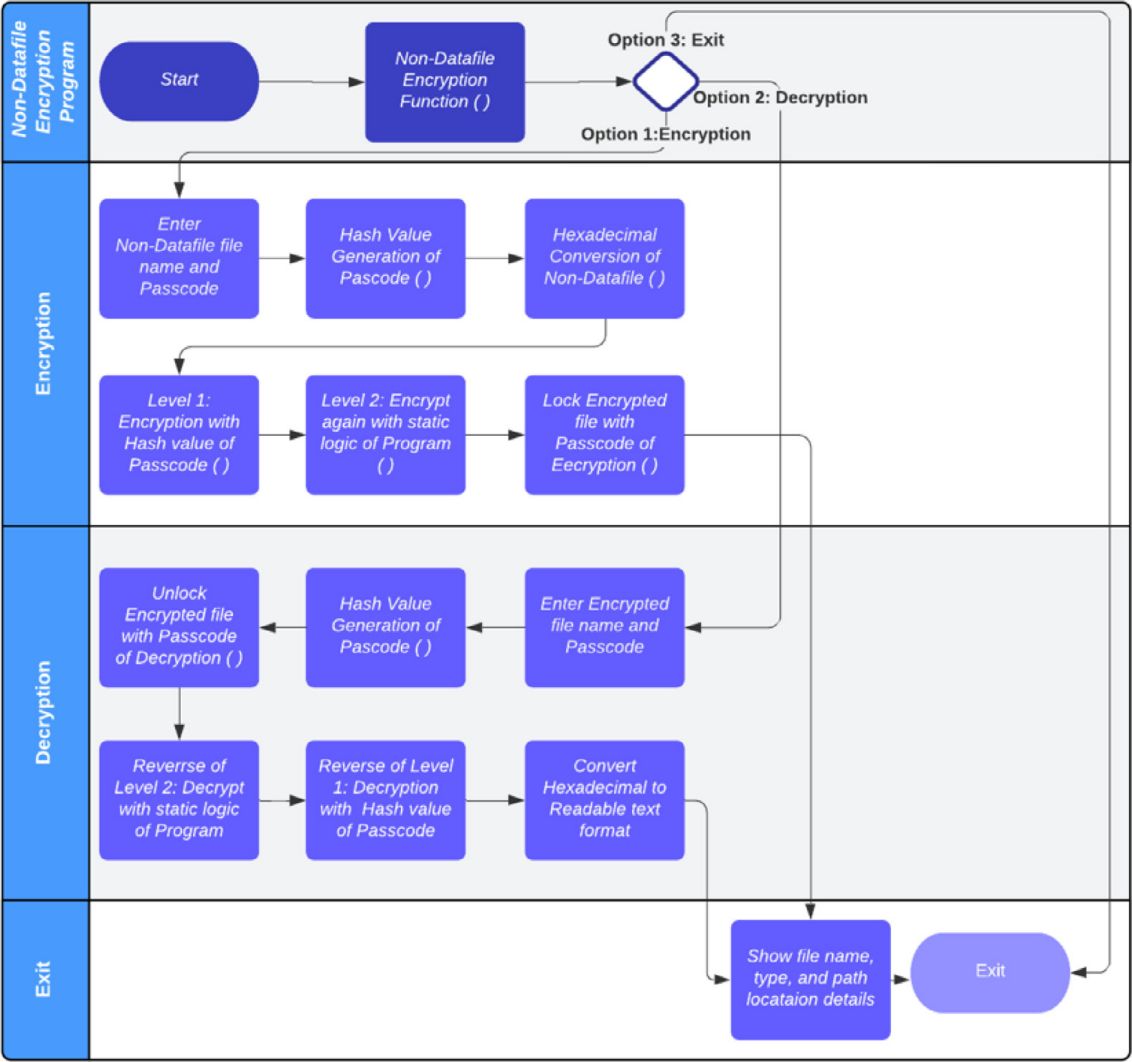
Process flow of non-datafile algorithm.

The process of encrypting the non-datafiles is shown in Fig. 2. In the process of initiating encryption, the user is presented with three options: encryption, decryption, and exiting from the program. Whenever the user decides to encrypt existing non-datafiles that are located on the database server, the file name and location must be provided as parameters as well as the passcode. It will trigger a specialized code and convert the passcode into numeral hash values. Consequently, the encryption program converts the text file into hexadecimal values and implements level one encryption using the hash value that is generated from the password. As a result, the encrypted content from level 1 is passed to level 2 encryption as an input, which triggers the second layer of encryption based on the algorithmic custom design logic. The implementation of this custom design logic will allow for the full control of encryption features to be chosen by the implementer. As a final step, the code turns the passcode into a password to ensure that the decryption fails without it.

If the user chooses the decryption option, then the program will be oriented towards decryption logic. The encrypted file name, location, and passcode must be passed as parameters. As a result, the passcode will be used to unlock encrypted file first in the system to be ready for next level of decryption. The reverse of level 2 encryption will then take place internally to complete level 2 decryption. Next, the decrypted information from level 2 would be passed on as input data for the level 1 decryption program. As a final step, the algorithm converts the non-datafiles into Hexadecimal format and later to human-readable text format.

When the user selects option 3, then the user's program will be terminated in the way he or she specified. On the other hand, after the completion of each phase of encryption or decryption, the user will be taken to the exit position when the file details are displayed.

### The pseudocode of non-datafile encryption algorithm

The algorithm of Non-Datafile Encryption (NDE) is presented as follows in pseudocode. As a result, the code is very feasible to replace a reader's customizable code with a new one. Thus, it would be up to the executioner to choose the encryption code.

**Table:** 

**Algorithm:**	**The Implementation of Non-Datafile Encryption (NDE) Algorithm**
Input:	Enter filename and Passcode
Output:	The Encryption or Decryption of file will be resulted.
Step 1:	Start
Step 2:	#!/bin/bash
Step 3:	Function: start_encryption () { read -p "Enter your Filename: " filename; read -p "Enter your Password: " passcodes; Hex decimal conversion: xxd -ps: Generate hash code; Level 1: Encryption: swap(hash); Level 2: Logic (character Swap); file password: add(passcode); echo "your encrypted file is Encrypted_file.txt"; }
Step 4:	Function: start_ decryption () { read -p "Enter your Filename: " filename; read -p "Enter your Password: " passcodes; Hex decimal conversion: xxd -ps: Generate hash code; file password: verify(passcode); Reverse of Level 2: Choice of Logic (character Swap); Reverse of Level 1: Decryption: swap(hash); echo "your encrypted file is Decrypted_file.txt"; }
Step 5:	Main Program () { echo "To do encryption, enter 1..." echo "To do decryption, enter 2..." echo "To do Exit, enter 3..." echo "Enter a number:" read num start case if($num==1) then call start_encryption; if($num==1) then call start_decryption; if($num !=1) && ($num !=2) echo "your entry does not match any of the conditions" end case;
Step 6:	end;

The following Table 1 gives the list of required non-datafiles from different databases products.

**Table 1:** The configuration files of databases.

Database Type	File name
MySQL	/etc/my.cnf
MySQL & Oracle	/etc/hosts
Oracle	$ORACLE_HOME/dbs/initora.ora
Oracle	$ORACLE_HOME/network/admin/tnsnames.ora
Oracle	$ORACLE_HOME/network/admin/listener.ora
Any Database	Important text files

### Implementation and execution of NDE algorithm

An NDE algorithm has been developed and achieved the desired results upon execution. This algorithm can be implemented on non-datafiles of any database product. The NDE algorithm and coding can be implemented on other database products like Oracle and DB2, etc. The file ‘my.cnf’ from the MySQL database has been selected to implement the algorithm in this section.

**Fig. 3:**
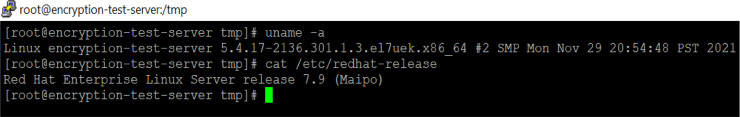
Server details.

The NDE algorithm has been implemented on a Linux machine as shown in Fig. 3. The server infrastructure does not have to be complex, just a simple database server is enough. MySQL was used as the database server in this article. Out of all the non-datafiles in the database, the parameter file was selected as a non-datafile. As the parameter file contains the username and password, it was chosen for the test cases to demonstrate the benefits of the proposed NDE algorithm and its execution capabilities.

**Fig. 4:**
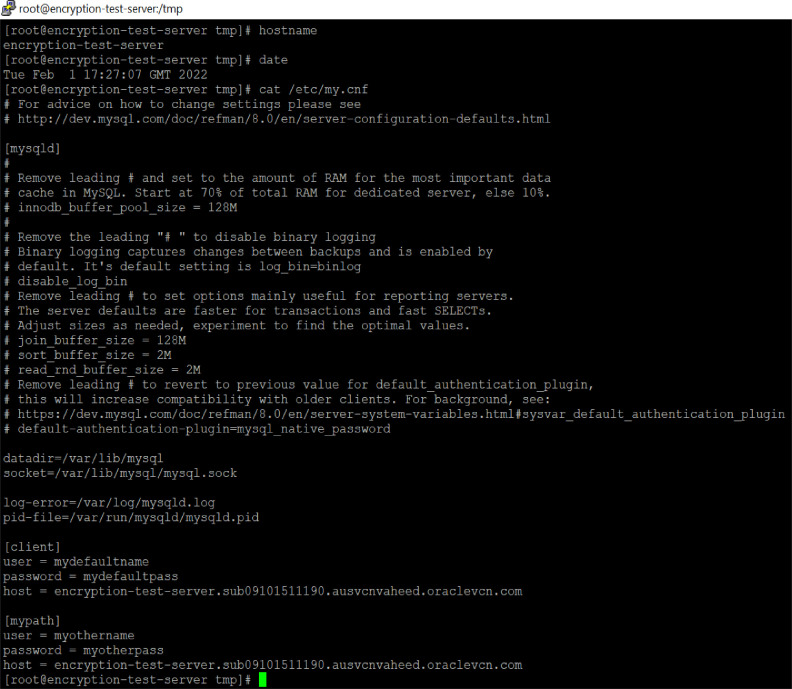
The content of original file.

The MySQL configuration file ‘/etc/my.cnf’ will exist in every MySQL database, as shown in Fig. 4. The content of the configuration file is always in text format and in some configurations, usernames and passwords are also included. A similar configuration setup is shown in Fig. 4. Here, usernames and passwords are exposed as text files. As a result of the absence of the NDE algorithm, the same files will be stored in remote storage backup locations such as tape drives or cloud buckets. An insider will have the opportunity to steal the usernames and passwords, as well as the database structures and store locations.

**Fig. 5:**
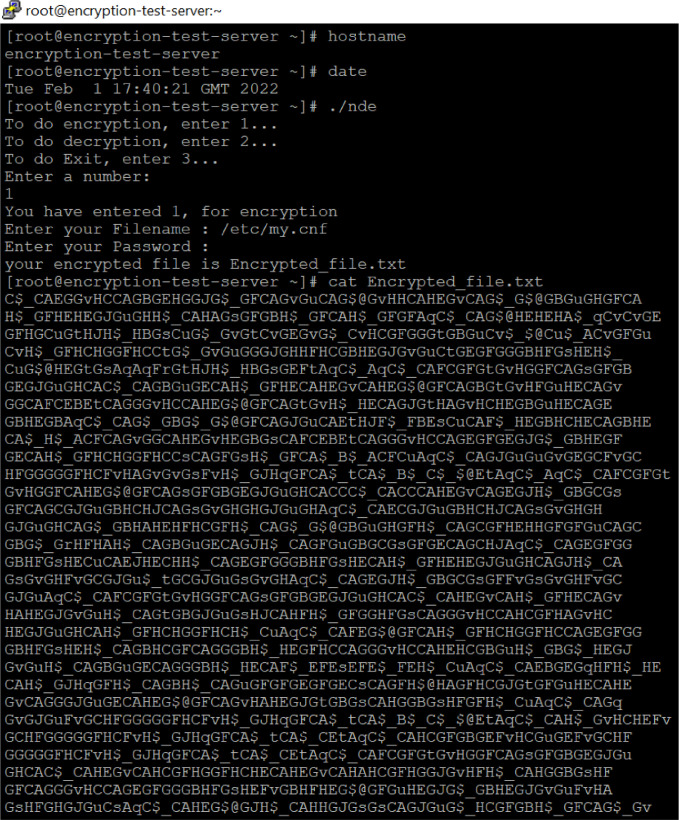
The content of encrypted non-data file.

As shown in Fig. 5, the encrypted file name will be displayed upon execution of the program. The location path would be exactly where it was initiated. The encrypted content of the can be seen in the Fig 5 for further observations. The proposed methodology code has been converted into a binary execution file named ‘nde’. The same ‘nde’ execution file has been executed as shown in Fig. 5. As mentioned in the process flow Fig. 2, the file name and passcode have been pushed as the parameters into the program. Upon completing the level 1 and level 2 encryptions as well as the file lock mechanism, the encrypted file name appears on the server screen. The encrypted text is displayed using the ‘cat’ command. The encrypted text prevents the insider from stealing valuable information from the backup locations. In addition, keeping such encrypted non-datafiles in a remote location where the owners don't have complete access to the data to make it always secure. An unauthorized individual will not be able to access this file until it is decrypted again by the authorized individual. Even if these encrypted files stolen by the insider, the encrypted content makes no sense to them.

**Fig. 6:**
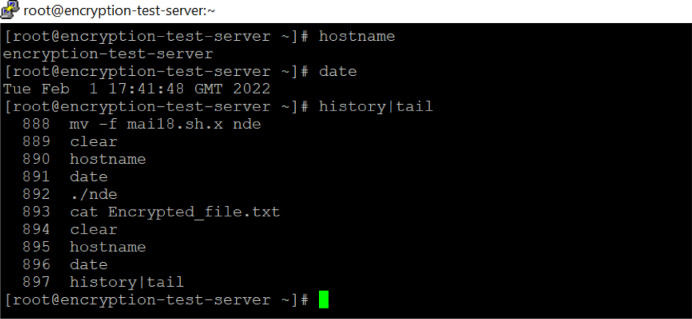
The history details of server.

According to Fig. 6, the history command with tail greps will provide the recently executed operating system level commands in the Linux systems. usually, this command is considered as proof of operations to show that there were no alterations or tampering in the result. It is proof of fact that the logic proposed on databases has been implemented and executed.

**Fig. 7:**
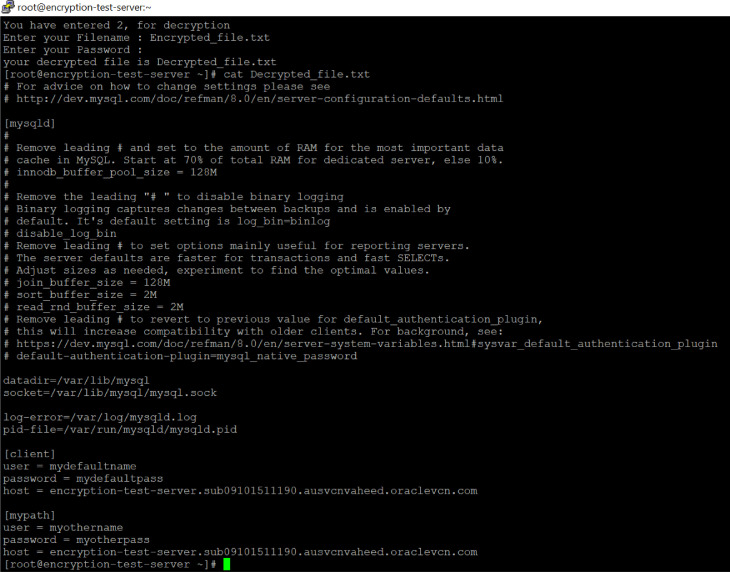
The content of decrypted non-datafile.

According to Fig. 7, decryption proceeds as follows. The process flow as depicted in Fig. 2; the authorized person will be asked to provide the name of the file which has been copied from remote backup storage locations. By sequentially entering the file name and password, the internal mechanism reverses the encryption level 2 and level 1 upon unlocking the file structure. When the program is about to exit from execution, it will display the decrypted file names on the screen. In Fig. 7, the reader can see the actual content of decrypted text file as it is before encryption.

## Method validation

As the text is encrypted, the algorithm converts it into a variety of symbols and alphanumeric characters. Each character that is recognizable by operating systems should have a numeric keycode. This paper follows the same logic to clarify the results using keycode comparisons. A keycode was substituted for encrypted codes to obtain an accurate transformation detail of pain text in non-datafiles. When the full results are presented, the scatterplots become clumsy. To get a better aesthetic view of the report, the first 200 characters and symbols are taken from the actual results.

### The Keys of non-datafile before applying the Encryption

**Fig. 8:**
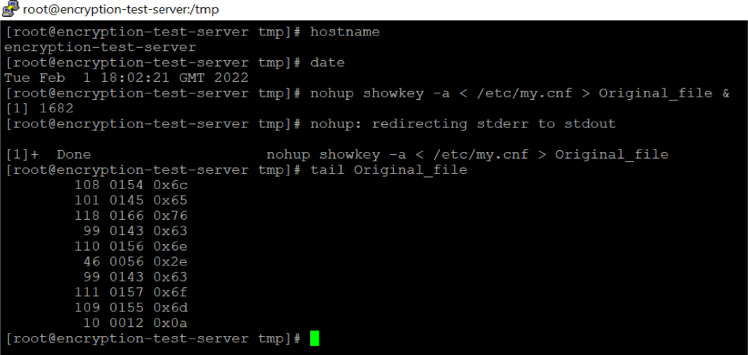
The process of keycode generation.

As shown in Fig. 8, In file ‘Original_file’ the first column shows the decimal value of the character. The second and third columns contain keycodes in octal and hexadecimal formats, respectively. Throughout the results section, the same keycode generation process is used to generate the keycodes. This is how the keycodes were derived from the original plain text file.

**Fig. 9:**
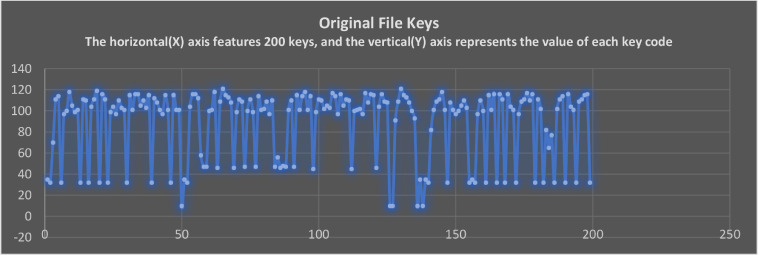
The keycodes of original file keys.

In Fig. 9, the keycodes for the original text are presented in scatter-plot format. Each scatter dot represents a keycode value of an original text character. The first dot in the graph represents the key value of 35. Similarly, the total sample of 200 key codes is displayed as dot in whole scatter plot.

### The keycodes of non-datafile representation after applying level-1 and level-2 encryption

**Fig. 10:**
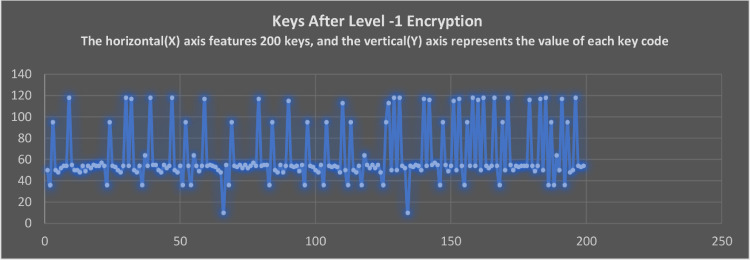
The keycodes of level-1 encrypted text keys.

Upon completing Level-1 encryption, Fig. 10 shows the keycodes for the encrypted text. NDE algorithm produces significant changes to the text. Almost all the hexadecimal text has been changed. The character representation will be changed to any other unknown symbol or character which is unreadable by humans. After the character representation is separated, the proposed hexadecimal algorithm performs the encryption. As an example, the letters ‘a’ and ‘b’ have the hexadecimal code ‘0 × 61’ and ‘0 × 62’ respectively. After the hexadecimal conversion, the cipher text logic will be applied to make the text of the character symbol invisible and unknown to the computer interpreter. The logic described in this paper makes it possible to blindfold even a computer interpreter from understanding the content.

**Fig. 11:**
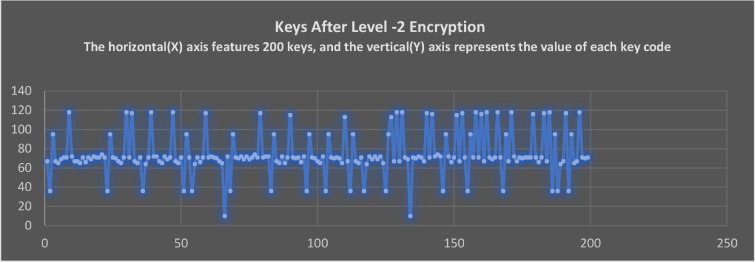
The keycodes of level-2 encrypted text keys.

As can be seen in Fig. 11, As a result of Level-1 encryption, the plain text will be converted into encrypted cipher text. These encrypted result sets will be encrypted again at Level-2 upon receiving from the Level-1. As shown in Fig. 11, the Level-2 encryption has effectively replaced all the keycodes from Level-1 encrypted text. By this step, the process of encrypting non-datafiles of databases has been completed. As a result of this encryption, the generated symbols will be unknown and unrelated to the previous text. By using these 2 levels of encryption, non-datafile content is transformed into code that a computer interpreter cannot understand.

### The Keycodes of Non-datafile representation after applying Level-1 and Level-2 Decryption

The Decryption takes place in a different way. Mathematically, the logic that makes A=B also gives B=A as a prediction. The proposed decryption logic does not allow this kind of prediction.

**Fig. 12:**
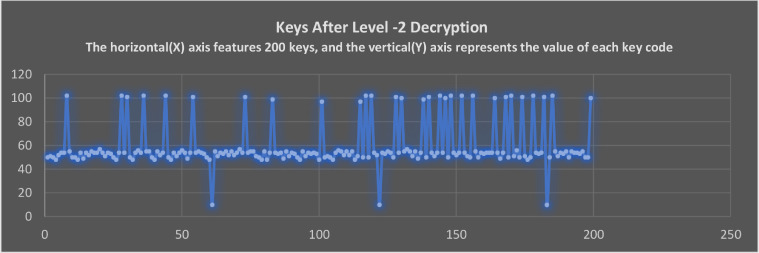
The keycodes of text after completion of level -2 decryption.

As shown in Fig. 12, the decryption begins with level-2 decryption. If the end-user/intruder enters a false process passcode at this point, the algorithm won't restrict the process or throw an error. Instead, it misleads whoever is performing the decryption by showing them more encrypted data. Here, the wrong passcode directs the program to generate a hash value for the wrong passcode and uses that hash value for the encryption. Ultimately the Intruder receives more encrypted data as the proposed program output.

The Level-2 decryption is applied to encrypted non-datafiles in Fig. 12. As a result of applied decryption logic, keycodes are transformed into different keycodes. Whenever decryptions are applied, the algorithm ensures that they do not return the same keycodes of encrypted text as it was in encryption phase. Logic such as this makes encryption algorithms more secure and less predictable to intruders. The Fig. 10 and Fig. 12 illustrate this representation. As the Fig. 10 is after applying the Level-1 encryption and Fig. 12 after decryption of Level -2.

**Fig. 13:**
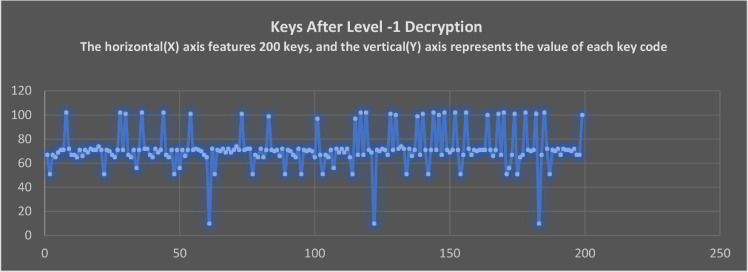
The keycodes of text after completion of level -1 decryption.

Upon applying the Level-1 decryption, Fig. 13 represents the keycodes of characters and symbols. As mentioned earlier, the codes are not transformed to their previous representations as before applying Level-1 encryption. This uniqueness arises from the swapping of a hash value generated from a passcode. The swap of hash values takes place at the end of the decryption process, as specified in the algorithm for decryption. Because decryption of Level-1 and 2 rely on a passcode, the decryption always generates unique keys which make the characterization process is difficult to predict.

**Fig. 14:**
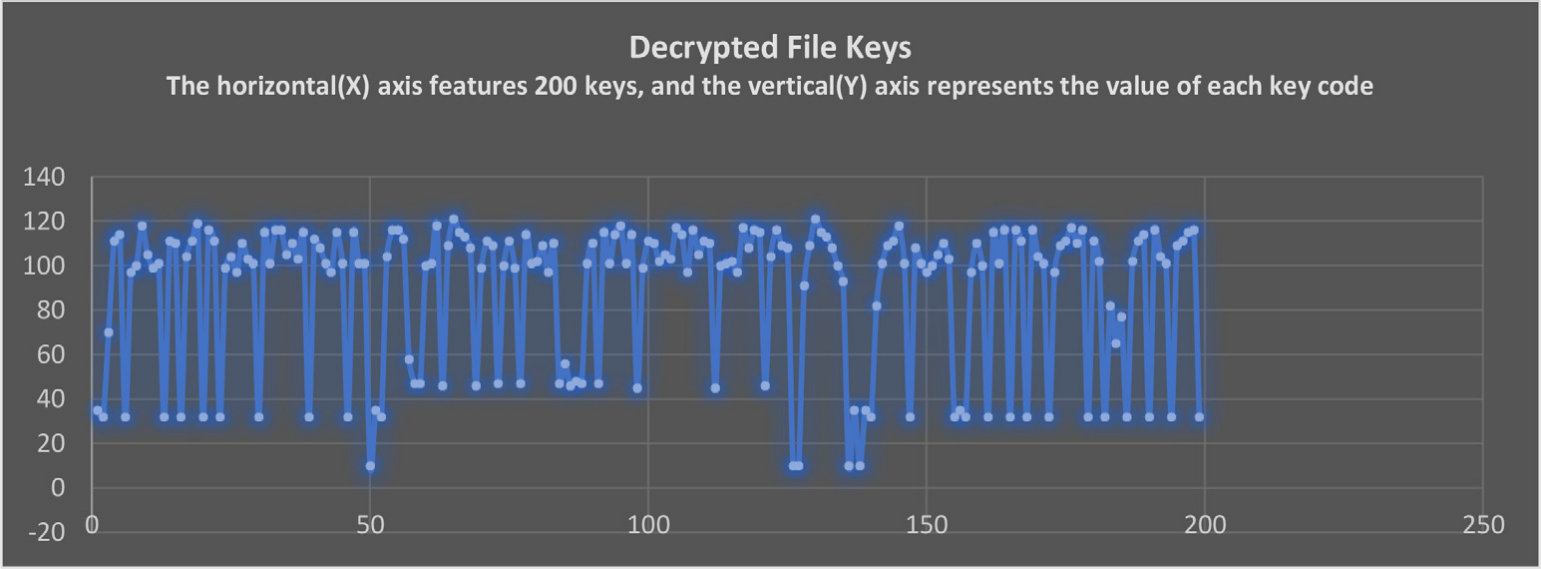
The keycodes of decrypted file keys.

Fig. 14 represents the actual keycode representation of the decrypted file upon completing the Level-1 and Level-2 decryption processes. The encrypted and decrypted texts do not match at any point during the encryption and decryption process. As an example, when a character A is converted into B by Level-1 encryption and then converted into C by Level-2 encryption, the representation is A=B=C. However, during the decryption process, the C never represents the B, and the B never matches the earlier encrypted symbols of A. A key strength of this algorithm is this. This is clearly visible in the graphs.

### Comparison of original and decrypted files keycodes

**Fig. 15:**
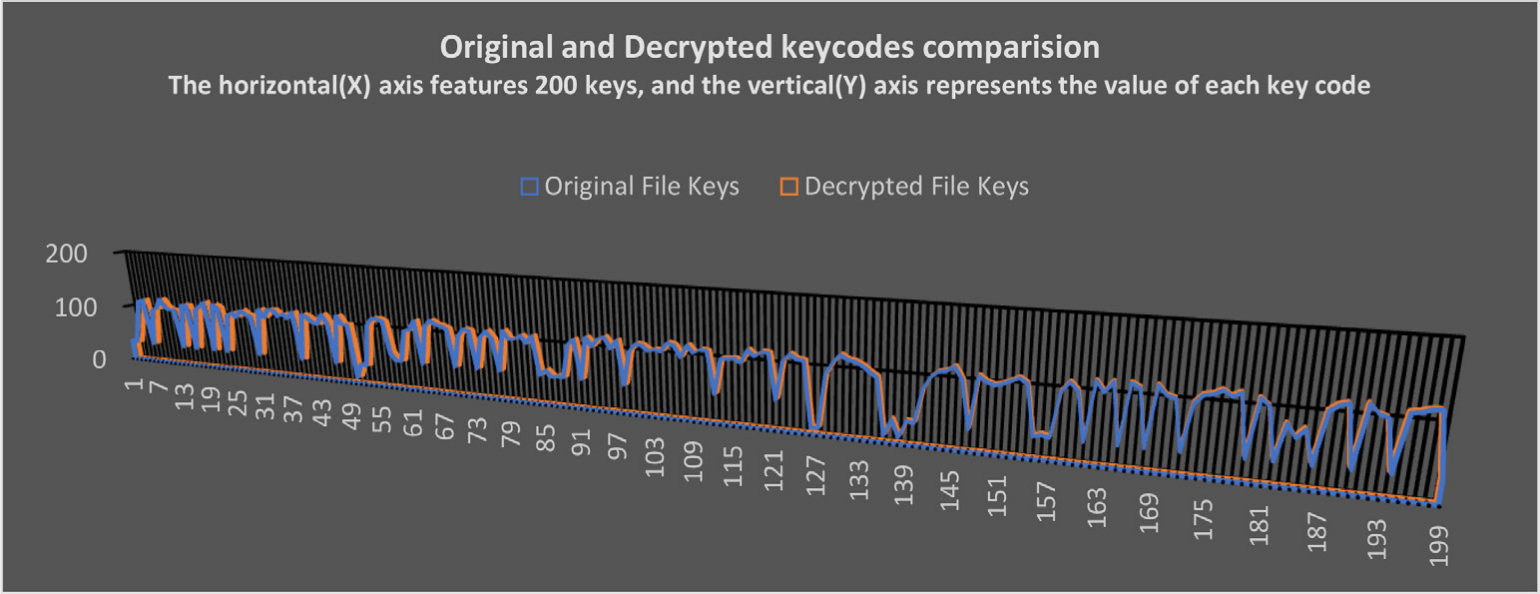
Comparison of keycodes of original and decrypted files.

Fig. 15 shows a comparison report of keycodes. It contains the key codes for the plain text before it is encrypted into cipher text. Upon completing all decryption cycles, these codes have been compared. Upon decryption, there is not a single mismatch found in the keycodes. This verifies that the results are accurate and authentic. The intruder gets more encrypted content each time he enters the wrong passcode. If an insider or intruder uses a long incorrect passcode, he receives even more encrypted data. As a result of entering the wrong passcode, these codes do not match, and this is expected behavior from the algorithm.

### Comparison of disk size occupancy of original and decrypted files

**Fig. 16:**
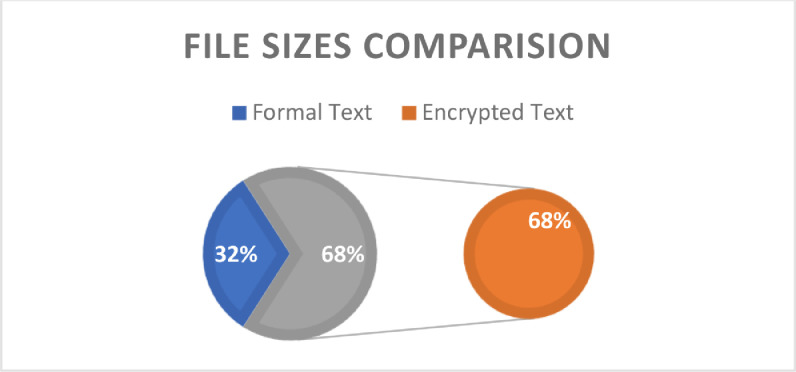
Representation of disk storage space utilization.

Fig. 16 shows a common instance of an increased file size after encryption. It is an expected characteristic of all encryption methods. In this paper, the same comparison was conducted to observe changes in the file occupied sizes before and after encryption. According to the NDE algorithm, the file size will occupy 68% of the storage space, while the unencrypted file will occupy 32%. Although there are two levels of encryption and a passcode lock implemented, the size difference between text and encrypted files is only 36%.

## Future work

The NDE algorithm opens many ways to progress in different directions. The datafiles of the database are currently using the Transparent Data Encryption (TDE) method which comes with an extra license. There are many companies that exist still not using the TDE due to the extra cost. The finding the means to apply the proposed methodology to the datafiles too can provide better cost management and goof flexibility in encryption.

The existing encryption algorithms always generate the same encrypted content output. The passcode parameter passing can change the encrypted output. However, it has dependency on the passcode, every time the passcodes must be changed otherwise it will generate the same encrypted content again. So, there is a necessity of passing one dynamic value along with a passcode to generate the unique encrypted content output every time. In future research, adding dynamic values like time variables along with passcode in the encryption process can make the logic to impossible to break.

The medium and small-level organizations keep the data in normal files and for them, it's difficult to manage licenses cost to keep their historical data securely. In future work, finding the way to apply the NDE algorithm to normal files along with non-datafiles will bring great benefit to all organizations and help to secure every individual personal information.

The implementation and results sections have proved the efficiency and flexibility of the algorithm. In future work developing the automatic process to encrypt and decrypt every saved file, helps to secure the data in small to medium organizations and every roadside merchandise shop. Every billing detail of customers can be secured permanently in the systems.

## Conclusions

The data of the databases are being transformed into encrypted statuses to protect them from insiders and intruders. To accomplish this same goal, most database products used Transparent Data Encryption on the database datafiles [24]. Although datafiles appear to be encrypted, it appears that other files are ignored. Further, encryption was not standardized for non-datafiles, so they had to rely on third-party tools to encrypt and secure them. The study proposes and explains a new encryption algorithm and the importance of encrypting non-datafiles. It also explains how non-datafiles that are not encrypted can threaten infrastructure and data. By implementing the proposed methodology, non-datafiles can be kept in encrypted format in remote backup storage locations. Furthermore, the proposed algorithm lets the owner of the files select the encryption logic of their choice, which has never been possible in existing algorithms. Hands-free, independent customization is made possible with the removal of the license managing cost burden. Encryption and decryption processes have been developed using two levels of encryption logic and a passcode combination lock, whereas the same two levels of reversing encryption and passcode are required to convert the data back into text format. By implementing the algorithm practically and examining its accumulated results, it is possible to illustrate the strength and benefits of the non-datafile encryption algorithm.

By turning the proposed methodology and algorithm into program code a new binary execution file ‘nde’ has been developed for Unix environments. The same ‘nde’ binary has been used throughout the results and discussions section. The further research is open to develop such an algorithm and code logic in windows environments.

## Declaration of Competing Interests

The authors declare that they have no known competing financial interests or personal relationships that could have appeared to influence the work reported in this paper.

## Data Availability

https://github.com/vaheedbashashaik/NDE_Algorithm.

## Appendix Supplementary materials

Image, application 1

Image, application 2
