# Supplementary material for: Flexible and cost-effective cryptographic encryption algorithm for securing unencrypted database files at rest and in transit
Source: MethodsX. 2022 Nov 11;9:101924. doi: 10.1016/j.mex.2022.101924 (PMC9692032; doi:10.1016/j.mex.2022.101924)
Supplement: Supplementary file 1 [file mmc1.docx]

**AIM:**

**To provide all supporting documents. The intention behind uploading the files in this format for better accessibility**

| **Purpose of File** | **File** |
| --- | --- |
| Original File content | **** |
| Original File Keycode for the text | **** |
| Text after applying Level-1 Encryption on Hexa-Decimal Text |  |
| Keycodes after applying Level-1 Encryption on Hexa-Decimal Text |  |
| Text after applying Level-2 Encryption on Hexa- Decimal Text |  |
| Keycodes after applying Level-2 Encryption on Hexa-Decimal Text |  |
| Text after applying Level-2 Decryption on Hexa- Decimal Text |  |
| Keycodes after applying Level-2 Decryption on Hexa-Decimal Text |  |
| Text after applying Level-1 Decryption on Hexa- Decimal Text |  |
| Keycodes after applying Level-1 Decryption on Hexa-Decimal Text |  |
| Decrypted File content |  |
| Decrypted File Keycode for the text |  |
| Executable Program ‘nde’ |  |

.

Thank you.
